# Neuropathological Mechanisms of Mild Traumatic Brain Injury: A Perspective From Multimodal Magnetic Resonance Imaging

**DOI:** 10.3389/fnins.2022.923662

**Published:** 2022-06-17

**Authors:** Yin Liu, Liyan Lu, Fengfang Li, Yu-Chen Chen

**Affiliations:** Department of Radiology, Nanjing First Hospital, Nanjing Medical University, Nanjing, China

**Keywords:** mild traumatic brain injury, functional MRI, arterial spin labeling, susceptibility weighted imaging, diffusion MRI

## Abstract

Mild traumatic brain injury (mTBI) accounts for more than 80% of the total number of TBI cases. The mechanism of injury for patients with mTBI has a variety of neuropathological processes. However, the underlying neurophysiological mechanism of the mTBI is unclear, which affects the early diagnosis, treatment decision-making, and prognosis evaluation. More and more multimodal magnetic resonance imaging (MRI) techniques have been applied for the diagnosis of mTBI, such as functional magnetic resonance imaging (fMRI), arterial spin labeling (ASL) perfusion imaging, susceptibility-weighted imaging (SWI), and diffusion MRI (dMRI). Various imaging techniques require to be used in combination with neuroimaging examinations for patients with mTBI. The understanding of the neuropathological mechanism of mTBI has been improved based on different angles. In this review, we have summarized the application of these aforementioned multimodal MRI techniques in mTBI and evaluated its benefits and drawbacks.

## Introduction

Mild traumatic brain injury (mTBI) is one of the most common neurological diseases, the incidence rate in North America and Europe is as high as 600/100,000, and two-thirds of cases occur in men (Maas et al., 2017; Carroll et al., 2020). mTBI is a serious social and economic challenge because it may be a risk factor leading to the consequences of cognitive decline, early dementia, and mental illness (McInnes et al., 2017). According to the American Association of Rehabilitation Medicine, mTBI is a traumatic physiological interruption of brain function with one or more of the following symptoms: Post-traumatic amnesia (PTA), loss of consciousness (LOC), neurotic disorder, and focal neurological deficit occurring after trauma, and the Glasgow Coma Scale (GCS) score was 13–15 within 30 min after trauma (Carroll et al., 2004). From the perspective of diagnosis, the evaluation of mTBI at present relies heavily on subjective clinical symptom reports (Samuelson et al., 2020). However, due to the different severity of clinical symptoms of mTBI, these reports are often unclear. In order to avoid the influence of subjectivity as much as possible, objective auxiliary inspection is particularly important.

With the development of magnetic resonance imaging (MRI) techniques, several novel multimodal imaging approaches can clearly show the potential neuropathological changes of mTBI, such as functional MRI (fMRI), arterial spin labeling (ASL) perfusion imaging, susceptibility-weighted imaging (SWI), and diffusion MRI (dMRI). These imaging techniques can be used to locate the injured brain functional area more accurately, so as to comprehensively evaluate the severity of the craniocerebral injury, greatly improve the early diagnosis accuracy of mTBI, and play a pivotal role in prognosis evaluation (Lunkova and Guberman, 2021). Nevertheless, these results have been blended with a more common application and a slower integration into the clinical setting. This paper has described the benefits and drawbacks of the aforementioned multimodal MRI techniques applied in mTBI published in recent years and the contributions from different angles to a deeper understanding of the underlying neuropathological mechanisms following mTBI.

## Methods

These studies were searched using the PubMed, with the filters set to the time period of the past 7 years from the moment when the paper was written (2015–2022) for task-based fMRI, for the past 6 years (2016–2022) for resting-state fMRI (rs-fMRI), and for the past 9 years (2013–2022) for ASL, SWI, and dMRI. The keywords were used for searching are as follows: “mTBI,” “fMRI,” “task-based fMRI,” “resting-state fMRI,” “ASL,” “SWI,” and “dMRI.”

### Task-Based Functional Magnetic Resonance Imaging

Task-based fMRI is based on the blood oxygenation level dependent (BOLD) method, in which the MRI signal difference between deoxyhemoglobin and oxygenated hemoglobin is extracted and monitored by gradient echo (GRE) sequence (Christen et al., 2013). Task-based fMRI activates the corresponding brain regions by completing specific experimental tasks, based on the activation of the neuronal response to stimulation and the increase of metabolic demand in the brain area, resulting in the change of local blood flow. These changes were recorded as BOLD signal changes during scanning. Brain function was evaluated by detecting and comparing BOLD signals. Working memory task (N-back), because of its advantages of easy presentation in the scanner and high sensitivity to changes in brain activity, has been widely used in the study of task-based fMRI. Other tasks included spatial navigation task, flanker task, distracted emotion assessment task, and visual tracking eye movement test.

In recent research, Hsu et al. (2015) found increased activation in bilateral frontal and parietal regions in the N-back study of patients with mTBI. van der Horn et al. (2017) found reduced neural activity of the medial prefrontal cortex in the N-back study of patients with mTBI. Moreover, Astafiev et al. (2015) observed reduced activation of the right anterior internal capsule and the right upper longitudinal tract in the visual tracking eye movement test in patients with mTBI. Combined with other recent similar studies (Saluja et al., 2015; Chen et al., 2016), it can be inferred that the compensation changes during task execution may be that some areas of the brain with increased activation compensate for those damaged areas showing decreased activation. In addition, it cannot be excluded that the increased or decreased activity of patients with mTBI during task execution may also be related to the cognitive needs of the task.

The dorsolateral prefrontal cortex (DLPFC) is one of the main components of the working memory network (Barch et al., 2003). Up to one-third of patients with mTBI demonstrated persistent cognitive deficits in executive function. Patients with mTBI had shown prefrontal cortex activity deficits during the performance of executive tasks that required active information maintenance and manipulation (Witt et al., 2010). Zhang et al. (2020) found that the weakening of DLPFC activation was the main brain region to distinguish patients with mTBI from healthy controls. Cognitive defects may be related to the weakening of DLPFC activation, which may be due to the decline in the recruitment ability of neural networks involved in controlling attention. It was found that the change in DLPFC activation was related to the participation of additional parahippocampal areas (Saluja et al., 2015; Holmes et al., 2019). During spatial working memory, the hippocampal prefrontal direct afferent pathway plays a key role in continuous updating of task-related spatial information. We suggested that the abnormal neural activity of the prefrontal cortex following mTBI likely influenced more basic, elemental cognitive processes that can still be thought of as “executive” in nature, not simply on difficult tasks requiring substantial mental effort or cognitive control (Witt et al., 2010).

In general, task-based fMRI has been widely used in patients with mTBI. Although its results are diverse, it has been proved to be an effective tool for diagnosing mTBI and predicting prognosis. However, its limitations include complex procedures and time-consuming, such as task development and patient training, making it difficult to popularize fMRI in clinical practice.

### Resting-State Functional Magnetic Resonance Imaging

Similar to task-based fMRI, rs-fMRI examines synchronous activation between different spatial regions based on the measurement of BOLD signal fluctuations to identify resting-state networks (RSNs) (Lv and Wang, 2018). Compared with task-based fMRI, this method focuses on the activation without task or stimulation. It is simpler and easier to study the spontaneous activity of the human brain in the resting state without the additional cooperation of subjects.

The default mode network (DMN) is one of the main RSNs in mTBI research. DMN may be related to memory consolidation, working memory, processing of significant emotional stimuli, and the interaction between emotional processing and cognitive function (Mohan et al., 2016). Madhavan et al. (2019) found in the longitudinal rs-fMRI study of patients with mTBI that when compared with the healthy control group, the DMN connectivity in the mTBI group was increased, and the longitudinal functional connectivity changes constituted a potential biomarker to predict the recovery curve and clinical outcome of mTBI. Similar results appeared in the study of van der Horn et al. (2017). However, in the study of D’Souza et al. (2020) the rs-fMRI results of the mTBI group within 7 days showed that DMN connectivity was decreased, suggesting that there was a negative correlation between network connectivity and the severity of symptoms after mTBI. A follow-up study of 6 months after injury showed that the network connection was increased and the severity of symptoms after concussion were improved. In addition, in the study of Zhang et al. (2021) about the effect of frontal white matter hyperintensities (WMH) on DMN connectivity after acute mTBI, it was found that frontal WMH volume was inversely proportional to DMN connectivity and directly proportional to post-traumatic symptoms, indicating that the WMH may be an effective biomarker for the diagnosis and prognosis of acute mTBI.

It should be noted that changes in the interaction between RSNs after mTBI may lead to a poor long-term prognosis. In the parallel study of task related network (TRN) and DMN related to attention activation, the change of interaction between DMN and TRN may lead to poor long-term memory consolidation (Lefebvre and D’Angiulli, 2019). In the study of Li et al. (2021) when compared with mTBI patients without post-traumatic headache (PTH), mTBI with PTH group showed four altered interactions, i.e., decreased interactions in salience network (SN)-sensorimotor network (SMN) and visual network (VN)-DMN pairs, increased interactions in SN-executive control network (ECN) and SMN-DMN pairs.

Moreover, mTBI is accompanied by changes in other RSNs, such as sensory motor, visual, auditory, and dorsal attention networks (Hou et al., 2019; Madhavan et al., 2019; D’Souza et al., 2020; Amir et al., 2021). The changes in these networks are also considered to be related to the manifestations of post-concussion syndrome (PCS) (Madhavan et al., 2019). Combined with the mTBI studies using rs-fMRI, there is a positive correlation between the changes in network connectivity and the manifestations of various PCSs. The changes in connectivity at an acute stage after injury are considered to be a predictor of subsequent cognitive difficulties (Palacios et al., 2017; Churchill et al., 2018; Li et al., 2019; Lu et al., 2019). In addition, previous studies used a graph theoretical approach to investigate alterations in brain network topology based on resting-state functional connectivity in patents with mTBI (Hou et al., 2019; Boroda et al., 2021; Sun et al., 2021). Moreover, several studies suggested that dynamic functional network connectivity (dFNC) can be used to identify optimal dFNC states for the classification of mTBI, which has shown potential as an important imaging modality for the development of mTBI biomarkers (Vergara et al., 2018; Li et al., 2021; Lu et al., 2022).

Resting-state fMRI study in mTBI shows the functional changes of the brain after mTBI and may predict the subsequent changes in cognitive ability. However, the complex post-processing process is the main factor that the sequence has not been included in clinical diagnosis. At the same time, the limitations of this method also include the low repeatability and specificity for rs-fMRI results.

### Arterial Spin Labeling

The cerebrovascular changes caused by mTBI play a critical role in the evolution of sequelae and brain repair after trauma. The main mechanism of ASL is the magnetic labeling of water protons in arterial blood and using them as endogenous tracers. It is an effective means for the non-invasive quantification of cerebral blood flow (CBF) (Bambach et al., 2022). ASL can be divided into two groups: Absolute CBF (aCBF) and relative CBF (rCBF). The aCBF value corresponds to the perfusion level of the region of interest (ROI) and is independent of other regions, while the rCBF value shows the change of ROI relative to other brain regions. Therefore, rCBF is more sensitive to focal CBF abnormalities, while aCBF value is specific to the whole brain.

Most mTBI-related studies using ASL found that when compared with the healthy control group, CBF in patients with mTBI showed a decrease (Peng et al., 2016; Wang et al., 2016, 2019; Churchill et al., 2017b) and further decrease in the first week after injury (Wang et al., 2016; Churchill et al., 2017b). Among them, at 24 h after injury, CBF in the mTBI group was decreased mainly in the right supplementary motor area (SMA) and pre-SMA regions (Wang et al., 2016). Within 24–48 h after injury, the decrease of CBF in the mTBI group was mainly distributed in the left inferior parietal lobe, the right superior marginal lobe, the right middle frontal gyrus, the posterior cingulate gyrus, the left occipital gyrus, and the thalamus (Wang et al., 2019). In the acute phase (within 72 h) and subacute phase (3 days–3 weeks), the CBF of the occipital lobe, the parietal lobe, the central area, the subcutaneous area, and the frontal lobe was decreased (Peng et al., 2016). Similarly, rCBF was decreased in the right insula, temporal lobe, and bilateral thalamus 1 month after injury (Bartnik-Olson et al., 2014; Meier et al., 2015). Moreover, the results of long-term longitudinal studies in the recovery period of mTBI also tend to have lower CBF or are statistically equivalent to the control group within 1 month to 1 year after injury (Churchill et al., 2019).

On the contrary, some studies showed that CBF was increased or did not change in patients with mTBI, which is inconsistent with the above studies. Doshi et al. (2015) found that rCBF in frontal lobe, occipital lobe, and left striatum was increased within 10 days after mTBI, but its sample size was small and its interpretation ability was limited. Stephens et al. (2018) investigated the adolescent athletes 2–6 weeks after injury. When compared with the control group, the rCBF of the left dorsal anterior cingulate cortex (ACC) and left insula was increased after 2 weeks. After 6 weeks, high rCBF was only present in the left dorsal ACC. One possible explanation may be that patients with more symptoms will have higher CBF values. Barlow et al. (2017) observed an overall increase of CBF in symptomatic mTBI and an overall decrease of CBF in asymptomatic mTBI when compared with the controls. Churchill’s study showed that for athletes with concussion, more serious symptoms were associated with increased CBF in the posterior cortex (Churchill et al., 2017a).

In addition, Peng et al. (2016) found that CBF in the temporal lobe and the marginal lobe was decreased in the acute and subacute phases but was recovered in the chronic phase (more than 3 months). Meier et al. (2015) found in the longitudinal study of mTBI that CBF in the right insular and upper temporal cortex was gradually recovered 1 day, 1 week, and 1 month after injury. Moreover, in athletes with slow recovery, CBF in the dorsal cortex of the insula was decreased 1 month after injury and was inversely proportional to the size of the initial mental symptoms. It is suggested that CBF has the potential to predict prognosis as a biomarker. Moreover, relevant studies have shown that there is a correlation between CBF changes and cognitive impairment after mTBI. Bai et al. (2019) showed that the increase of CBF in the posterior parietal cortex (PPC) of men with mTBI when compared with healthy men can predict the worse cognitive performance of male patients. The decline of CBF was associated with lower clinical cognitive assessment scores 24–48 h after injury in athletes with sport-related concussion (Wang et al., 2019).

However, although most ASL studies try to control the differences between subjects in data analysis, such as age, gender, genetics, biomechanics, and predisease psychological function, which may affect CBF before and after the disease. The impact of these factors has not been clarified in the existing studies of ASL on mTBI. Moreover, ASL has the disadvantages of low signal-to-noise ratio, poor time resolution, and post-labeling delay, which makes ASL need to be combined with other MRI techniques in order to reflect a greater value in the clinical diagnosis of mTBI.

### Susceptibility-Weighted Imaging

Susceptibility-weighted imaging is a full flow compensated 3D GRE sequence, which is particularly sensitive to microbleeds, venous blood, and iron levels (Haller et al., 2021). The main principle of SWI sequence is to use the magnetism of iron, especially in different hemoglobin states, to cause magnetic field distortion, affect T2* relaxation time and phase data, and superimpose the collected and processed phase information on the amplitude information, to enhance the difference of magnetic sensitivity between different tissues, and form the final SWI diagram. Paramagnetic substances (vein, bleeding metabolites, calcium, and iron deposition, etc.) show the loss of signal of SWI.

Microbleeding is a sign of traumatic axonal injury (TAI) and one of the main signs of mTBI. Einarsen et al. (2019) detected TAI lesions on SWI scan at 3 and 12 months after injury in the mTBI group. Studies have shown that SWI can detect more microbleeds than CT and conventional MRI (de Haan et al., 2017) and can show more clearly the boundary and scope of microbleeds (Tao et al., 2015). Early detection of microbleeds in mTBI helps to predict prognosis in the presence of PCS (Beauchamp et al., 2011; Geurts et al., 2012). Studerus-Germann et al. (2018) found that the presence of acute cerebral tissue microbleeds detected by SWI was associated with poor cognitive outcomes and persistent PCS in patients with mTBI. Wang et al. (2014) compared the difference in microbleeding lesions on SWI between the depression group and the non-depression group 1 year after mTBI and found that the number and volume of microbleeding lesions in the depression group were greater than those in the non-depression group.

Scholars have studied the location of microbleeds in different brain regions. Wang et al. (2014) found that the difference in the number and volume of microbleeding lesions was only in the frontal, parietal, and temporal lobes. In the chronic phase of mTBI, microbleeds mainly occurred in the frontal and temporal lobe regions, and the prognosis of poor function was only related to the number and scope of microbleeds in the temporal cortex (de Haan et al., 2017). In addition, in the study of evaluating the changes in brain iron deposition level in patients with chronic mTBI through SWI, Lu et al. (2015) found that the angular radian value of the mTBI group (related to excessive iron deposition) was significantly higher in the head of caudate nucleus, hippocampus, thalamus, lenticular nucleus, right substantia nigra, red nucleus, and corpus callosum than other parts and found that the decline of cognitive ability in patients with mTBI was negatively correlated with the angular radian value of right substantia nigra.

Due to the biophysical properties of microbleeds, SWI is less sensitive to hyperacute bleeding (Környei et al., 2021). SWI showed that the sensitivity of traumatic microbleeds was positively correlated with the severity of injury (Trifan et al., 2017), and traumatic microbleeds were not detected in some mild patients (Toth et al., 2013). In addition, SWI may overestimate the microbleeding focus due to its high spatial resolution, and other factors cannot be excluded, such as the existence of microbleeding focus caused by hypertension and vascular malformation, and some structures show susceptibility effects that can simulate microbleeding, such as iron deposition in the basal ganglia and artifacts at the bone air interface. Nevertheless, the above studies still show the superiority of SWI when compared with other techniques in detecting small bleeding lesions. SWI is a complementary and valuable imaging technique of mTBI.

### Diffusion Magnetic Resonance Imaging

Previous studies have found that mTBI often leads to axonal shear damage to WM microstructure (Manley and Maas, 2013). Diffusion tensor imaging (DTI) is a non-invasive magnetic resonance diffusion imaging technique that is commonly used to examine the microstructure and pathological changes of cerebral WM fibers (Assaf and Pasternak, 2008). DTI quantifies the water diffusion pattern related to brain structure. Based on the assumption that water molecules follow Gaussian distribution, the characteristics of proton diffusion are described by using apparent diffusion coefficient (ADC), mean diffusivity (MD), and partial fractional anisotropy (FA). The main quantitative index is FA, and the higher FA value reflects the diffusion of water molecules along an axis, indicating that the axons are large in diameter, dense in axons, and high in myelination. The lower FA value reflects the diffusion of water molecules in all directions, which may indicate histopathological changes, such as axonal degeneration, demyelination, and increased edema.

The acute phase change of DTI index after injury varies greatly in different studies, and the expression of FA value increase or FA decrease is different (Yallampalli et al., 2013; Eierud et al., 2014; Churchill et al., 2017b; Palacios et al., 2020; Kim et al., 2022). Churchill et al. (2017b) found that when compared with the control group, the FA value was decreased 1–3 days after injury and increased 5–7 days after injury. The main change areas include the left upper radiation crown, corpus callosum, and the right junction of posterior thalamic radiation. In the study of Palacios et al. (2020), 2 weeks after injury, the FA of the knee and body of the corpus callosum, the anterior and posterior limbs of the internal capsule, the radiation anterior crown, the anterior radiation of the thalamus, the external capsule, and the cingulate gyrus in the mTBI group were lower than the healthy controls. In the chronic phase after mTBI, most reports believe that FA values are reduced (Palacios et al., 2020; Oehr et al., 2021; Stenberg et al., 2021).

In addition, DTI plays a role in predicting cognitive changes and recovery after mTBI. Veeramuthu et al. (2015) performed DTI scanning in patients with mTBI at an average of 10 h after trauma and evaluated their neuropsychological performance at an average of 4.35 h after the complete recovery of Glasgow Coma level. It was found that the FA value was negatively correlated with cognitive task performance in the hyperacute phase. Strauss et al. (2016) found that the increase of the FA value of the left frontal lobe and the left temporal lobe was related to the better performance of attention-related tasks by comparing the DTI scanning results on the 16th day and 1 year after injury. Meier et al. (2016) found that the increase in FA in the left upper longitudinal tract was associated with a longer recovery time in the post-acute and subacute concussion stage. Prior studies have demonstrated that patients with mTBI experience microstructural damages in the long-distance WM connections, which disrupt the functional connectome of large-scale brain networks that support cognitive function (Jia et al., 2021; Zhang et al., 2021; Palacios et al., 2022). Jia et al. (2021) provided novel evidence for functional and structural alterations of WM networks in patients with mTBI. Importantly, the convergent damage of the inferior fronto-occipital fasciculus network might imply its crucial role in our understanding of the pathophysiology mechanism of patients with mTBI. Furthermore, a multimodal MRI strategy was applied to capture dynamic topological features of both structural and functional connectivity networks, provide more sensitive detection of altered functional connectivity networks from its anatomical backbone, and identify novel biomarkers of mTBI (Wang et al., 2021).

However, the limitations of DTI are also obvious. Traditional DTI indicators, such as MD and FA, represent the basic statistical description of diffusion and do not directly correspond to the biophysical parameters of the underlying tissue. In addition, DTI relies on the assumption of Gaussian diffusion in a single microstructure, so it is not sensitive to the complexity of the WM microstructure. Therefore, although FA can easily detect WM damage in the context of mTBI, due to its poor specificity, it cannot be used to distinguish different forms of WM neuropathology and may lead to conflicting results in previous DTI studies.

More and more studies are applying novel dMRI methods on mTBI. Among them, the role of neurite orientation dispersion and density imaging (NODDI) has been used. NODDI uses the high-performance magnetic field gradient of an MRI scanner, which can realize the diffusion weighting factor much higher than DTI standard, so as to detect the more complex non-Gaussian characteristics of WM diffusion (Zhang et al., 2012). Palacios et al. (2020) found that NODDI was more sensitive to WM microstructure changes caused by mTBI than DTI by comparing the DTI and NODDI inspection results of long-term longitudinal WM changes after mTBI. The same comparison was confirmed in the experiment of Oehr et al. (2021).

## Conclusion

Taken together, the damage mechanism of patients with mTBI has a variety of pathological processes. The above methods elaborate the neuropathological mechanism of mTBI from different angles, but there is a lack of general neuroimaging diagnostic methods, indicating that a variety of imaging methods need to be used in combination with neuroimaging examination for patients with mTBI. In addition, there exist several problems in previous studies, such that the sample size of patients with mTBI is relatively small and the type of mTBI is various due to different injury mechanisms. Large-scale long-term longitudinal research is required to draw systematic and standardized research conclusions to verify the common applicability and practicability in the clinical settings.

## Author Contributions

YL checked the references and wrote the manuscript. LL and FL helped review and revise the manuscript. Y-CC contributed to the discussion and manuscript revision. All authors contributed to the article and approved the submitted version.

## Conflict of Interest

The authors declare that the research was conducted in the absence of any commercial or financial relationships that could be construed as a potential conflict of interest.

## Publisher’s Note

All claims expressed in this article are solely those of the authors and do not necessarily represent those of their affiliated organizations, or those of the publisher, the editors and the reviewers. Any product that may be evaluated in this article, or claim that may be made by its manufacturer, is not guaranteed or endorsed by the publisher.
